# High polymerase ε expression associated with increased CD8+T cells improves survival in patients with non-small cell lung cancer

**DOI:** 10.1371/journal.pone.0233066

**Published:** 2020-05-20

**Authors:** Kyueng-Whan Min, Wan-Seop Kim, Dong-Hoon Kim, Byoung Kwan Son, Young Ha Oh, Mi Jung Kwon, Hye Seung Lee, Seung Eun Lee, In Ae Kim, Ji-Yong Moon, Kyoung-Yeon Kim, Jung-Hoon Park

**Affiliations:** 1 Department of Pathology, Hanyang University Guri Hospital, Hanyang University College of Medicine, Guri, Gyeonggi-do, Republic of Korea; 2 Department of Pathology, Konkuk University Medical Center, Konkuk University School of Medicine, Seoul, Republic of Korea; 3 Department of Pathology, Kangbuk Samsung Hospital, Sungkyunkwan University School of Medicine, Seoul, Republic of Korea; 4 Department of Internal Medicine, Eulji Hospital, Eulji University School of Medicine, Seoul, Republic of Korea; 5 Department of Pathology, Hallym University Sacred Heart Hospital, Hallym University College of Medicine, Anyang, Gyeonggi-do, Republic of Korea; 6 Department of Internal Medicine, Konkuk University Medical Center, Konkuk University School of Medicine, Seoul, Republic of Korea; 7 Department of Internal Medicine, Hanyang University Guri Hospital, Hanyang University College of Medicine, Guri, Gyeonggi-do, Republic of Korea; 8 Macrogen Inc., Seoul, Republic of Korea; Inha University Hospital, REPUBLIC OF KOREA

## Abstract

DNA replicase polymerase ε (POLE) is critical in proofreading and correcting errors of DNA replication. Low POLE expression plays a pivotal role in accumulation of mutations and onset of cancer, contributing to development and growth of tumor cells. The aim of this study is to reveal the survival, alternative genes and antitumoral immune activities in non-small cell lung cancer (NSCLC) patients with low POLE expression and provide treatment strategies that can increase their survival rates. This study investigated the clinicopathologic parameters, various tumor-infiltrating lymphocytes (TILs), endogenous retrovirus, molecular interactions and in vitro drug screen according to POLE mutation/expression in 168 and 1,019 NSCLC patients from the Konkuk University Medical Center (KUMC) and the Cancer Genome Atlas, respectively. We identified mutations of 75 genes in the sequencing panels, with POLE frame shift p.V1446fs being the most frequent (56.8%) in KUMC based on 170 targeted sequencing panels. Mutant and high expression of POLE correlated with favorable prognosis with increased TILs and tumor mutation burden, compared with wild type and low expression of POLE. We found specific molecular interactions associated with cell cycle and antigen presentation. An in vitro drug screen identified dasatinib that inhibited growth of the NSCLC cell line with low POLE expression. POLE could contribute to the future development of anticancer drugs for patients with NSCLC.

## Introduction

Lung cancer is the most frequently diagnosed major cancer (approximately 2,090,000 global cases in 2018) and the most common cause of cancer mortality worldwide (1,760,000 deaths in 2018) in the World Health Organization data. It has traditionally been classified as either non-small cell lung cancer (NSCLC) or small cell carcinoma (SCC) according to histological criteria. According to the National Comprehensive Cancer Network Clinical Practice Guidelines in Oncology, early NSCLC requires surgical resection, but advanced NSCLC and SCC are treated with systemic therapy, which is a complete cure option [1] However, nearly 50% of patients will relapse, usually within the first year after initial treatment [2,3]. Therefore, molecular studies to identify mechanisms and biomarkers for this group of patients are currently being pursued. Published data have shown that epidermal growth factor receptor (EGFR) and anaplastic lymphoma kinase (ALK), molecules for first-line target therapy, improve prognosis for patients with lung cancer. Recent studies supported PD-L1, a targeted protein in immunotherapy for malignant melanoma, as a target for immunotherapy of NSCLC [4,5].

DNA replicase polymerase ε (POLE) plays a role in proofreading and correcting errors of DNA replication [6], a crucial process for avoiding mutation accumulation in dividing cells [7]. Cancer progression partially depends on DNA replication proofreading as well as the mismatch repair system that may affect proliferation and growth of tumor cells. The combination of mutations in mismatch repair and DNA polymerase results in an extremely rapid accumulation of mutations and onset of cancer [7].

POLE mutations have been found in various types of malignant neoplasms such as endometrial, colorectal, brain, stomach, breast, and pancreatic cancers [8–10]. NSCLC comprises 6% to 8% of somatic mutations in the proofreading exonuclease domain of POLE [11,12]. In the Catalogue of Somatic Mutation in Cancer (COSMIC) database, POLE mutants predominantly include missense substitutions (82.33%), followed by synonymous substitutions (11.73%), nonsense substitutions (3.61%), and frameshift deletions/insertions (2.45%) in 44 types of malignancy [13]. Although several mutant variants of POLE have been discovered, there are 252 substitutions in which the nucleotide change in the POLE mutant is unknown. Previous studies revealed that POLE mutants are associated with a high number of single-nucleotide variants (> 100 mutations/Mb) known as a “hypermutated” phenotype [9,14]. Hypermutated POLE variants are related to favorable prognoses in endometrial and colorectal cancers as well as high-grade glioma [15]. However, mRNA expression alterations and clinicopathological differences according to POLE mutation are still unclear in NSCLC.

**Fig 1:**
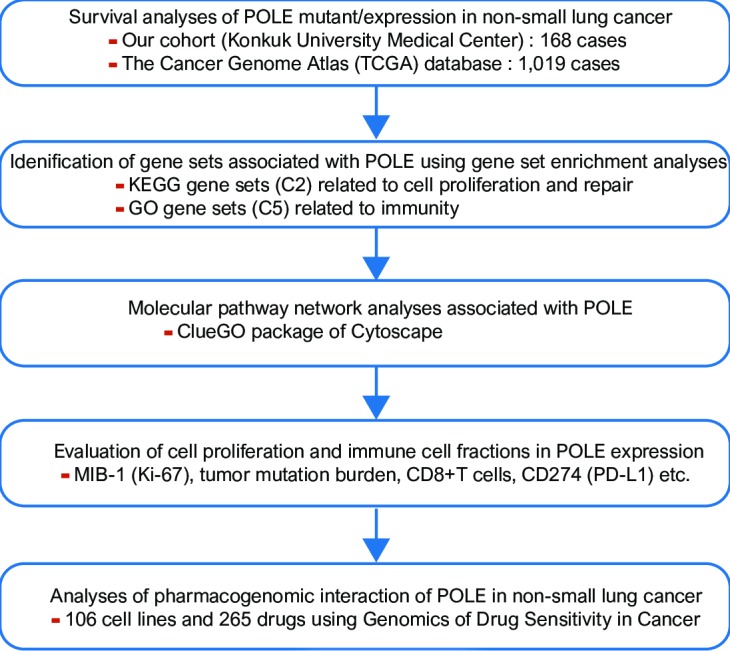
A schematic diagram depicting the analysis pipeline of this study.

This study aimed to determine if the exonucleolytic proofreading activity of POLE contributes to survival and growth of NSCLC and to analyze its prognostic value in both the Konkuk University Medical Center (KUMC) cohort and the Cancer Genome Atlas (TCGA) database [12]. We further aimed to identify gene sets related to POLE expression using gene set enrichment analysis (GSEA) [16] and pathway network analyses [17,18]. Distributions of tumor-infiltrating lymphocytes (TILs) and endogenous retroviruses (ERVs) expression were analyzed according to POLE expression [19]. Using the Genomics of Drug Sensitivity in Cancer (GDSC) and the Catalogue Of Somatic Mutations In Cancer (COSMIC) databases, we performed high-throughput drug sensitivity screening in lung cancer cell lines according to POLE expression (Fig 1) [20,21].

## Materials and methods

### Patient selection and sequencing

This study comprised 168 patients who underwent surgery for NSCLC at KUMC in Korea between 2005 and 2016. The Reporting Recommendations for Tumor Marker Prognostic Studies (REMARK) criteria were followed throughout this study [22]. The inclusion criteria were: 1) patients with histopathological evidence of primary NSCLC confirmed by pathologists and known clinical outcome; and 2) patients who did not receive chemotherapy. Cases with unavailable paraffin blocks or inadequate clinical history were excluded. Using the Custom Cancer Panel (Agilent Technologies, Inc., Santa Clara, California, USA) after DNA isolation from formalin-fixed, paraffin-embedded (FFPE) samples, we sequenced 170 cancer-related genes to identify genetic mutations (S1 File).

This study (involving human participants) was approved by the Ethics Committee of KUMC, Seoul, Republic of Korea (KUH1210049) and was performed according to the ethical standards of the Declaration of Helsinki, as revised in 2008. The review conducted by our institutional review board confirmed that informed consent was not necessary for this study. The archival data were fully anonymized before the beginning of the research and were waived the requirement for informed consent by the Institutional Review Board (IRB) of KUMC.

### GSEA, CIBERSORT, and molecular network analysis from the TCGA database

We obtained a total of 1,019 NSCLC cases comprised of 502 lung adenocarcinomas and 517 squamous cell carcinomas with known mRNA expression and mutations from the TCGA database [23]. For detection of significant gene sets, GSEA (version 3.0) was performed for 17,810 gene sets in the Molecular Signatures Database (MSigDB 6.2) from the Broad Institute at MIT [16]. Specific gene sets (186 KEGG sets, 20 immune sets) were used to identify those associated with POLE. For this analysis, 1,000 permutations were utilized to calculate the *p* values, and permutation type was set to phenotype. Significant gene sets were defined as follows: false discovery rate (FDR) < 0.2; family wise-error rate (FWER) < 0.4; *p* < 0.05.

We analyzed tumor-infiltrating lymphocytes (TILs) using deep learning-based lymphocyte classification with Convolutional Neural Networks (CNNs) in whole-slide image analysis and immune subtype using CIBERSORT and Kallisto software and algorithms [19,24–26]. Endogenous retroviruses (ERVs) were detected using fast gapped-read alignment with Bowtie 2 and the annotation of known expressed elements [27,28].

Pathway network analyses were based on identified POLE-associated genes using Cytoscape (version 3.7.2) network visualization software. To visualize the biological relevance of POLE and its relevant elements, we performed functional enrichment analyses using ClueGO, an application within the Cytoscape software [17,18].

### Data extraction from the GDSC and COSMIC databases

Drug screening was performed using the GDSC and COSMIC datasets, large-scale cancer cell line and drug response databases of 1,065 cancer cell lines and 265 anticancer drugs, respectively. In 106 NSCLC cell lines, anticancer drug sensitivity was measured by natural log half-maximal inhibitory concentration (LN IC50). A drug was defined as an effective POLE-targeted drug when the LN IC50 decreased in NSCLC cell lines with low POLE expression but increased in those with high POLE expression.

### Statistical analysis

Student’s t-test and/or Pearson’s correlation were used to examine the differences or relationships among continuous variables. In the KUMC cohort, disease-free survival (DFS) was defined as survival from the date of diagnosis to recurrence/new distant metastasis, and overall survival (OS) was defined as survival from the date of diagnosis to cancer-specific death. Survival curves were generated using the Kaplan–Meier method and compared using the log rank test. A two-tailed *p* value < 0.05 was considered statistically significant. All data were analyzed using R packages and SPSS statistics (version 25.0, SPSS Inc., Chicago, IL, USA).

## Results

### Clinical manifestations of POLE in non-small cell lung cancer

**Fig 2:**
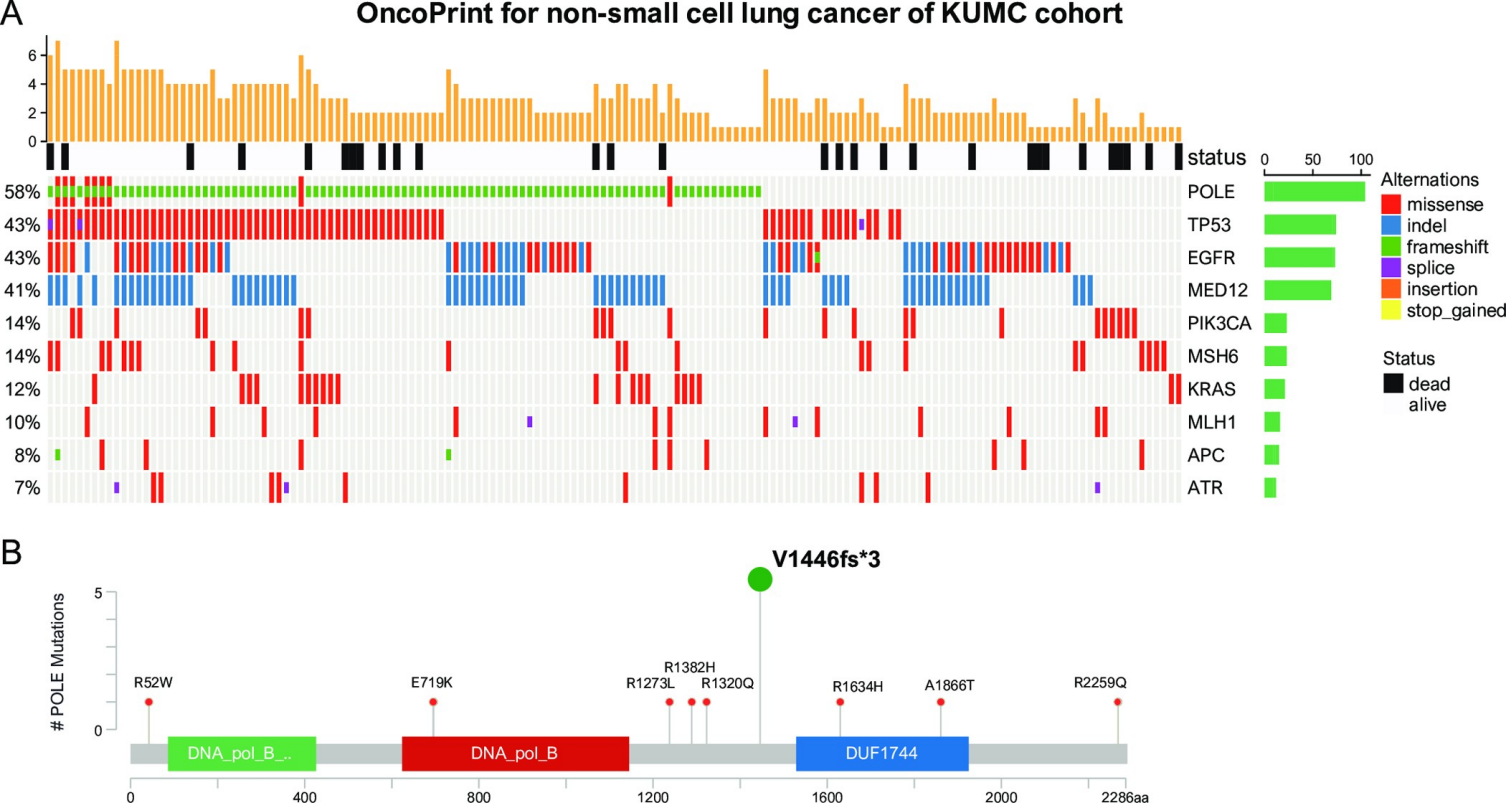
Schematic overview of the overall mutation profiles of 168 non-small cell lung cancers, followed by targeted NGS and analysis. (A) Each column represents an individual case. The top panel shows patient survival. The bottom panel shows the distribution of mutations. The 6 mutation types are distinguished by color. The right panel represents the overall frequency and the cancer hotspot mutation frequency. (B) Locations of POLE mutations.

In the KUMC cohort, POLE mutants were evaluated in 168 primary cancers (137 cases of adenocarcinoma and 31 cases of squamous cell carcinoma). Of the 75 identified mutation profiles, the top 10 are as follows: POLE (57.74%), EGFR (44.64%), TP53 (42.86%), MED12 (41.07%), PIK3CA (13.69%), MSH6 (13.69%), KRAS (12.50%), MLH1 (9.52%), ATR (9.52%), and APC (8.33%) (Fig 2A) (S1 Table). Therefore, the study focuses on the clinical significance and biological functions of POLE, as it has been shown to be the most frequently mutated. The most common type of POLE mutation was p.V1446fs *3 (Deletion–Frameshift) (Fig 2B) (S2 Table).

**Fig 3:**
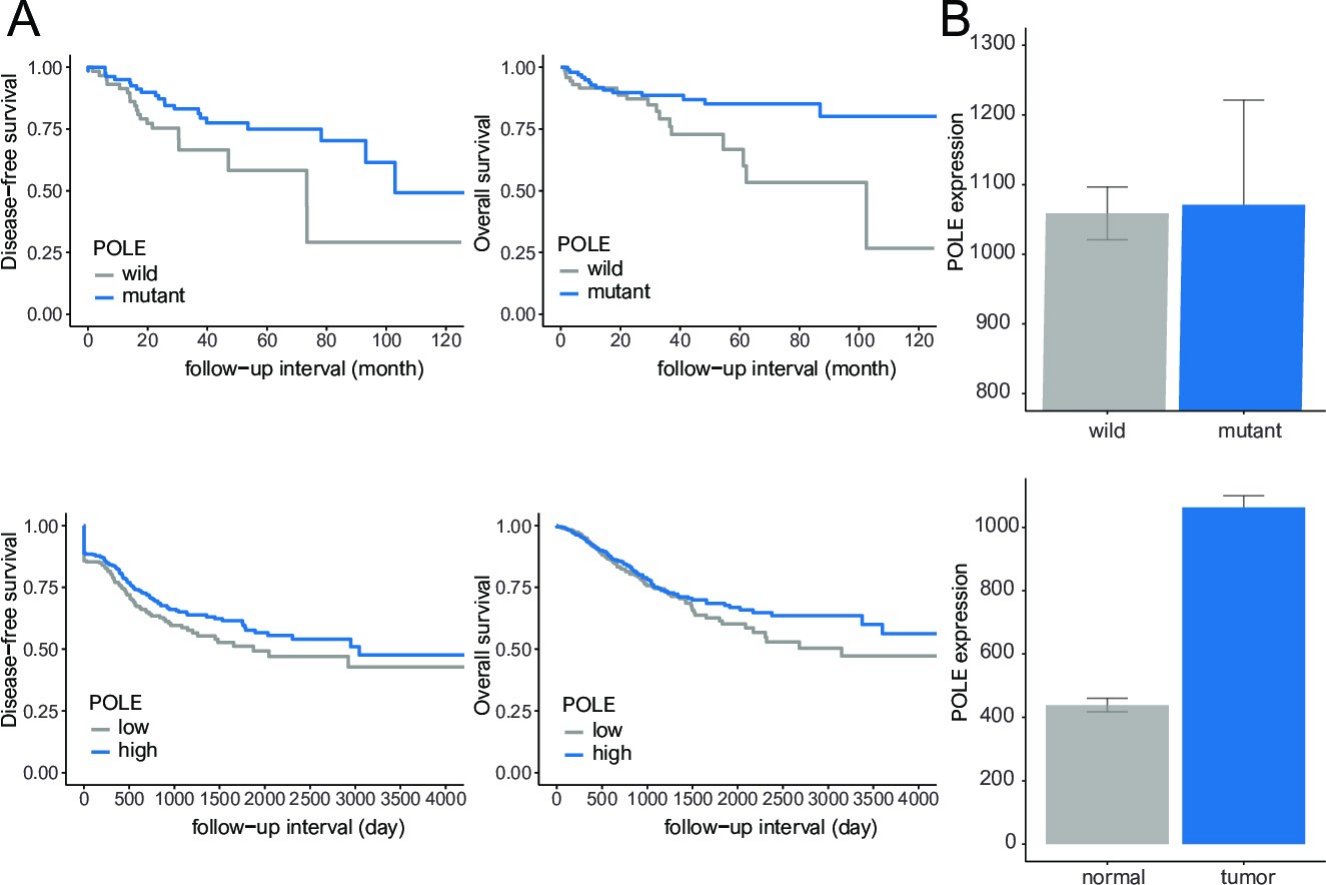
Survival analyses and bar plots of POLE. (A) Mutations (top) and high expression (bottom) of POLE were associated with favorable disease-free and overall survival (all p <0.05). (B) POLE was highly expressed in POLE mutants and tumors compared with wild-type and normal tissue.

In the KUMC cohort, POLE mutants were significantly associated with favorable DFS and OS (*p* < 0.05). In NSCLC from the TCGA, POLE revealed high expression in the mutant-type compared to the wild-type. NSCLC showed higher POLE expression compared to normal tissue (*p* < 0.001). High POLE expression significantly correlated with better DFS and OS compared to low POLE expression (*p* < 0.05) (Fig 3).

### Gene set enrichment analysis, anticancer immune response, and endogenous retrovirus of POLE

**Fig 4:**
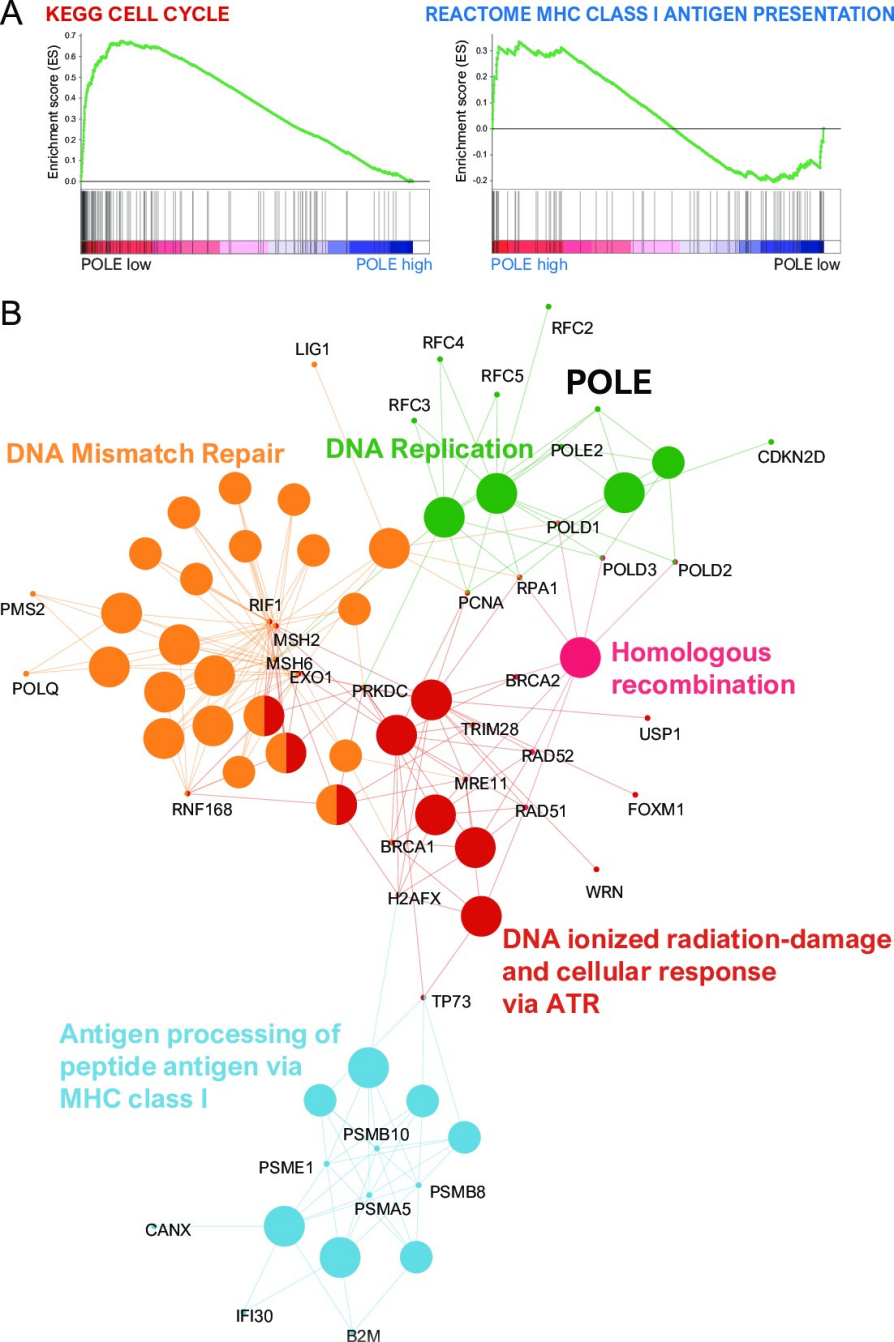
(A) Low POLE-dependent cell cycle gene sets (left) and high POLE-dependent MHC class I antigen presentation (right) using gene set enrichment analysis. (B) Grouping of networks based on functionally enriched GO terms and pathways. Functionally grouped networks are linked to their biological function, where only the most significant term in the group is labeled. There was a direct or indirect linkage among POLE, DNA replication, DNA mismatch repair, homologous recombination, DNA ionized radiation-damage and cellular response via ATR and antigen processing and peptide antigen via MHC class I.

We performed GSEA to identify various gene sets associated with POLE. We found 11 gene sets (KEGG CELL CYCLE, KEGG SPLICEOSOME, KEGG HOMOLOGOUS RECOMBINATION, KEGG DNA REPLICATION, KEGG MISMATCH REPAIR, KEGG PROGESTERONE MEDIATED OOCYTE MATURATION, KEGG BASE EXCISION REPAIR, KEGG LYSINE DEGRADATION, KEGG OOCYTE MEIOSIS, KEGG BASAL TRANSCRIPTION FACTORS, and KEGG RNA DEGRADATION) associated with low POLE expression reflecting an unfavorable prognosis. High POLE expression reflecting a favorable prognosis were linked to two gene sets (REACTOME ANTIGEN PRESENTATION FOLDING ASSEMBLY and PEPTIDE LOADING OF CLASS I MHC) (S3 and S4 Tables). In the analyses of molecular interaction pathway networks, POLE was directly linked to DNA replication and was indirectly linked to DNA mismatch repair, homologous recombination and DNA ionized radiation-damage and cellular response via ATR. The immune reaction relevant to antigen processing and peptide antigen via MHC class I was indirectly linked by the various genes associated with POLE (Fig 4).

**Fig 5:**
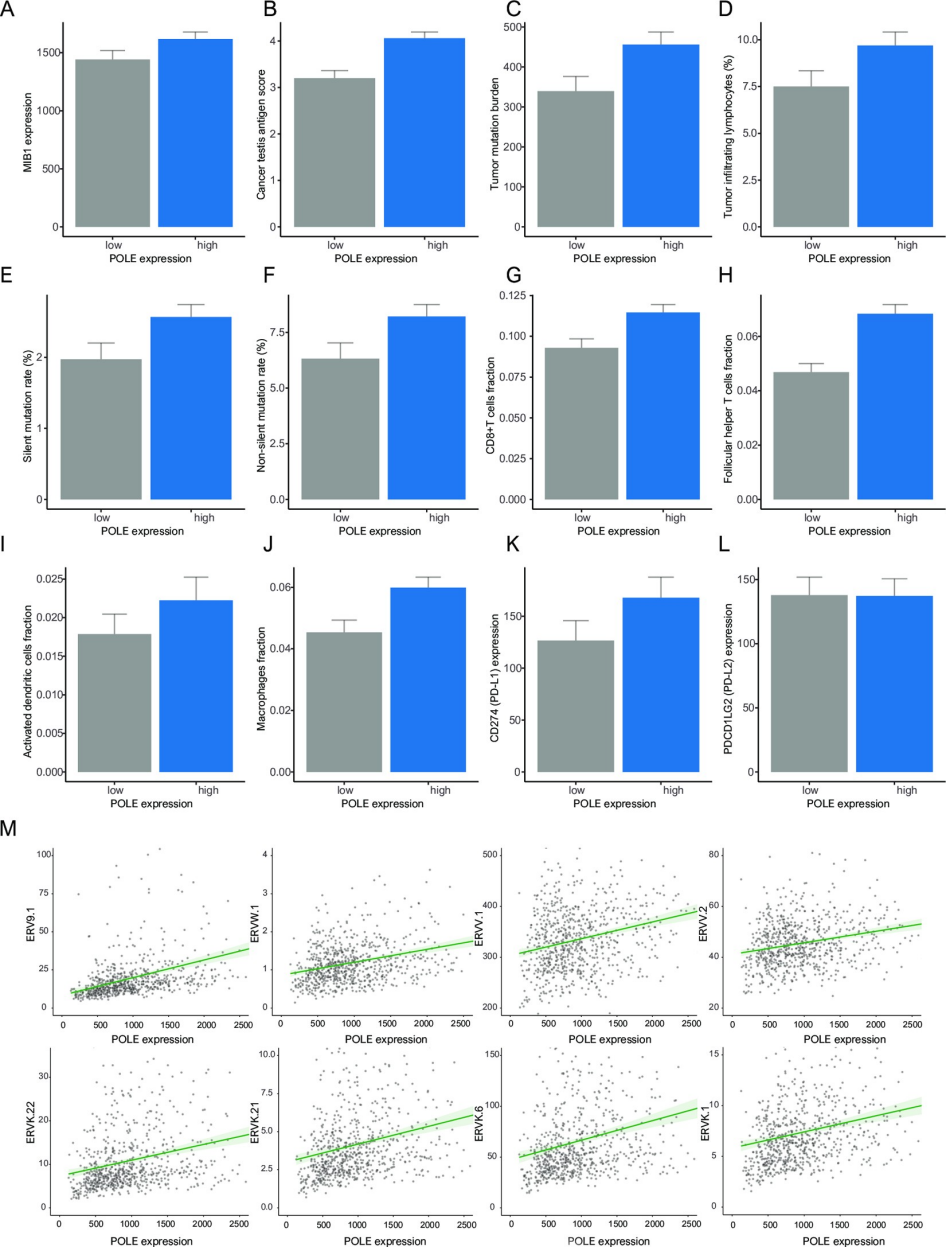
Bar plots of POLE and the following parameters: (A) MIB-1 (Ki-67), (B) Cancer testis antigen, (C) Tumor mutation burden, (D) Tumor-infiltrating lymphocytes, (E) Silent mutation rate, (F) Non-silent mutation rate, (G) CD8+T cells, (H) Follicular helper T cells, (I) Activated dendritic cells, (J) Macrophage, (K) CD274 (PD-L1), (L) PDCD1LG2 (PD-L2). (M) Pearson’s correlations showing positive correlation between POLE and endogenous retrovirus (ERV): ERV9-1, ERVW-1, ERVV-1, ERVV-2 (top); ERVK-22, ERVK-21, ERVK-6, ERVK-1 (bottom).

High POLE expression was associated with increased MIB-1 (encoded Ki-67), a proliferation marker, as well as high expression of cancer testis antigens (CTAs), tumor mutation burden (TMB), silent mutation rate, and non-silent mutation rate (all *p* < 0.001). In analyses of immunity, high POLE expression was correlated with high TIL percentage, CD8+ T cells, follicular helper T cells, activated dendritic cells, and macrophages (all *p* < 0.001). High POLE expression was related to increased CD274 (encoded PD-L1) (*p* < 0.001). There was no significant relationship between PDCD1LG2 (encoded PD-L2) and POLE expression. High POLE expression was associated with 36 types of ERVs (ERV9-1, ERVW-1, ERVV-1, ERVV-2, ERVK-22, ERVK-21, ERVK-6, ERVK-1, etc.) as ancient acquired elements (p < 0.05) (Fig 5) (S5 Table).

### Drug screening of lung cancer cell lines

**Fig 6:**
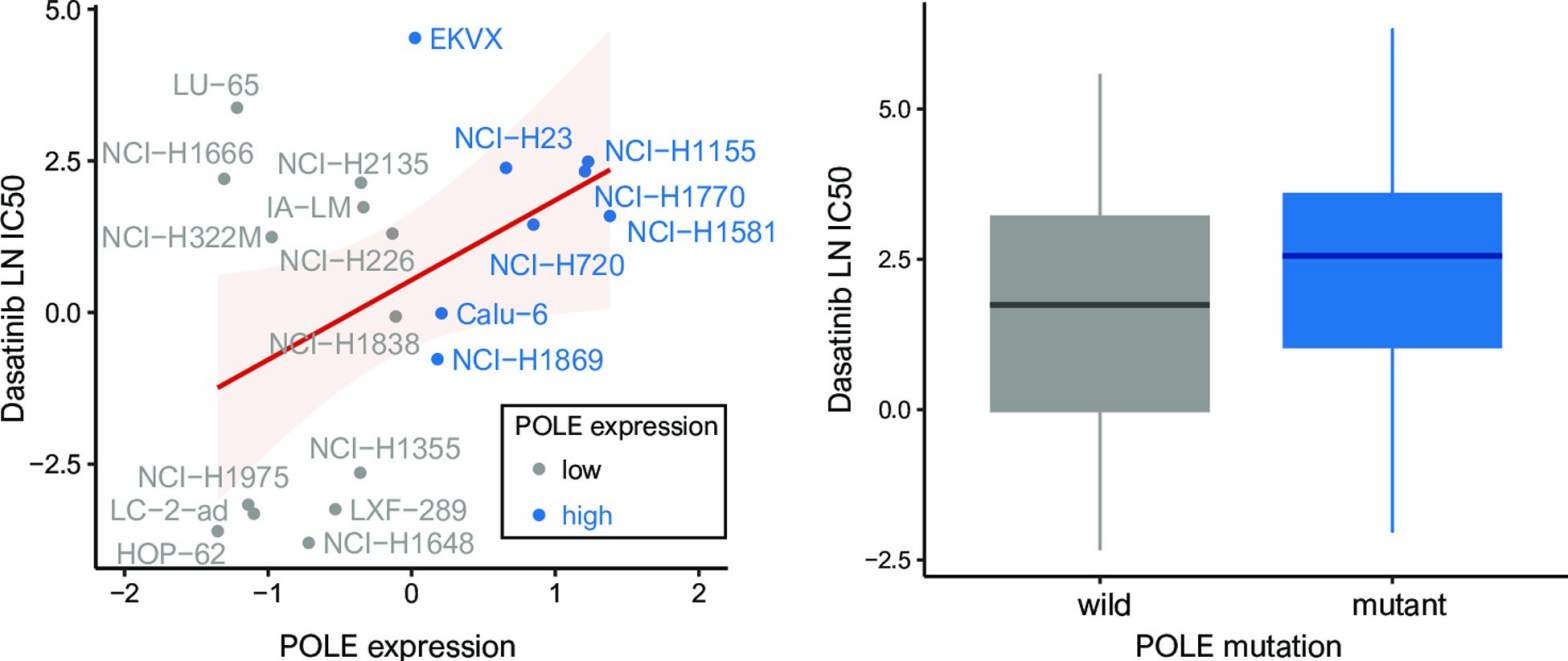
Pearson’s correlations showing the natural log half-maximal inhibitory concentration (LN IC50) of dasatinib, the most sensitive anticancer drugs against lung cancer cell lines with low POLE expression (right) and wild type of POLE (left).

We found 14 anticancer drugs that most effectively reduced the growth of cell lines with low POLE expression: Dasatinib, SCH772984, Trametinib, PD0325901, Ulixertinib, Pelitinib, Selumetinib, CI-1040, P22077, VX-11e, SB505124, ARRY-520, UMI-77, Refametinib (all *p* < 0.05). Among fourteen drugs, 3 drugs such as Dasatinib, Pelitinib, CI.1040 were highly sensitive to cell lines with wild type of POLE (Fig 6).

Dasatinib, most sensitive drug, was commonly founded to cell lines with low POLE expression and wild type of POLE.

## Discussion

In cancer progression, loss of DNA polymerase proofreading activity may contribute to favorable conditions for tumor growth and subsequent spread and metastasis [6]. This study demonstrates that POLE mutants are associated with better DFS and OS in patients with NSCLC compared to wild-type. In TCGA, the POLE mutation itself may affect high POLE expression. POLE mutations resulting in high POLE expression were associated with favorable DFS and OS in NSCLC. Thus, POLE may be helpful in predicting clinical outcomes that play an important role in inhibiting cancer progression. Unexpectedly, our results showed that POLE expression were higher in primary cancer than in healthy tissue. It can be deduced that error-prone DNA replication in rapidly growing cancer cells may induce high POLE activity. Notably, high POLE expression was related to increased cell proliferation, which would predict progressive disease but was also associated with hypermutation, which could recruit immune cells such as antigen-presenting cells, CD8+ T cells, and follicular helper T cells. This implies that the apparent improved survival time in patients with high POLE expression is more influenced by antitumoral immune cells than by cell proliferation. Interestingly, there was a positive relationship between POLE and CD274, suggesting that adaptive immune resistance by high immune response [29]. The adaptive immune resistance are thought to be the group that are largely responding to anti-PD-L1 therapy [30].

Previous studies demonstrated that POLE mutants are associated with improved clinical outcomes in various types of malignancy that were explained by enhanced host immune responses and increased sensitivity to anticancer drugs in colorectal and endometrial cancers [10,31]. However, the interactive molecules and pathways involved that are specific to these POLE mutations have not been clearly shown. The results presented here, based on integrated analyses of genetic alterations, demonstrated that POLE expression may be increased in its mutant form in NSCLC. In GSEA, low POLE expression was linked to cell cycle gene sets, while high POLE expression was linked to MHC class I antigen presentation gene sets. In analyses of functionally grouped networks, POLE was directly associated with DNA replication and was indirectly related to antigen processing of peptide antigen via MHC class I, as well as DNA mismatch repair, homologous recombination and DNA ionized radiation-damage and cellular response via ATR. These results show that co-expression of POLE and immune-associated gene elements may influence recruitment of immune cells to tumor sites in NSCLC.

An increased immune reaction is crucial to completely eliminate cancer cells. The significant correlation between POLE and TILs suggests that POLE is involved in DNA proofreading/repair as well as recruitment of immune cells to mediate cancer surveillance. Interestingly, high POLE expression was associated with increased ERVs, a remnant of an ancient retroviral infection, which induced immune responses by unleashing ERV expression from epigenetic restrictions, a process also known as “viral mimicry” [32]. In other words, analogous to an infecting agent, the ERV-derived nucleic acids are sensed in the cytoplasm and activate innate immune responses that drive the tumor cell into apoptosis. Thus, elevated ERVs associated with high POLE expression have other effects resulting in inhibited cancer growth.

In pharmacogenomic screens of 108 lung cancer cell lines, we identified anticancer drugs responsive to cell lines with low POLE expression. Of the effective anticancer drugs, dasatinib is an oral, small-molecule, Src family kinase (SFK) inhibitor that suppresses NSCLC progression via BRAF, PDGFR, ABL1, and DDR2 [33–38]. A study by Murakami et al. demonstrated that SFK inhibitors overcome multiple epidermal growth factor receptor (EGFR) family tyrosine kinase inhibitors and Afatinib resistance through suppression of the SFK/FAK-AKT axis and/or SFK/FAK-ERK axis [39]. Moreover, EMT-dependent and EGFR-tyrosine kinase inhibitor (TKI)-resistant NSCLC may benefit from combination therapy of Erlotinib with dasatinib [40,41]. Therefore, dasatinib treatment may contribute to improved therapeutic strategies for resistance to targeted drugs in NSCLC with low POLE expression.

This study has several limitations that should be acknowledged. First, because this is a retrospective study, and in silico analyses of POLE did not show sustained relationships over time, it is difficult to come to a definitive conclusion. Second, experimental data allowing for novel biological insights of mutation (p.V1446fs *3)-induced overexpression of POLE were not shown in the KUMC cohort. Further in vitro and/or in vivo studies may be necessary to clarify molecular mechanisms. Third, pharmacokinetics in lung cell lines may be highly heterogenous in NSCLC patients with various pharmacodynamics affected by disease status, microenvironments, and immunities.

## Conclusions

This study demonstrated that high POLE expression associated with increased cell proliferation, somatic hypermutation, and elevated ERV expression produced a favorable prognosis in patients with NSCLC. This implies that high POLE expression could enhance recruitment of TILs through hypermutation and/or ERVs rather than effects on cell proliferation. The increased CD8+T cells and CD274 overexpression in patients with POLE overexpression could be the result of adaptive immune resistance, which is an indicator of anti-PD-L1 therapies. Dasatinib, an identified anti-cancer drug was response against NSCLC cell lines with low POLE expression which could be treatment options for improving the survival of patients with NSCLC. In addition, our analytic workflow of POLE will contribute to designing future experimental studies and drug development in patients with NSCLC.

## Supporting information

S1 File (DOCX)

S1 Data (TXT)

S1 Table Gene mutation profiles in non-small cell lung cancer in the KUMC cohort. (DOCX)

S2 Table Protein sequence changes in the POLE mutants in the KUMC cohort. (DOCX)

S3 Table Eleven KEGG gene sets associated low POLE expression levels (TCGA data). (DOCX)

S4 Table Immune gene sets associated with high POLE expression levels (TCGA data). (DOCX)

S5 Table Pearson's correlation between high POLE expression levels and endogenous retroviral elements. (DOCX)
