# Supplementary material for: High polymerase ε expression associated with increased CD8+T cells improves survival in patients with non-small cell lung cancer
Source: PLoS One. 2020 May 20;15(5):e0233066. doi: 10.1371/journal.pone.0233066 (PMC7239475; doi:10.1371/journal.pone.0233066)
Supplement: S1 File — (DOCX) [file pone.0233066.s001.docx]

**Targeted Sequencing**

Using the Custom Cancer Panel (Agilent Technologies, Inc., Santa Clara, California, USA) after DNA isolation from formalin-fixed, paraffin-embedded (FFPE) samples, 170 cancer-related genes were sequenced (ABL1, ABL2, AKT1, AKT2, AKT3, ALK, APC, AR, ARAF, ASXL1, ATM, ATR, AURKA, AURKB, AURKC, AXL, BAP1, BCL2, BRAF, BRCA1, BRCA2, BRD2, BRD3, BRD4, CBFB, CCND1, CCND2, CCND3, CCNE1, CDH1, CDK12, CDK4, CDK6, CDKN1A, CDKN1B, CDKN2A, CDKN2B, CDKN2C, CEBPA, CHEK2, CREBBP, CRKL, CSF1R, CTNNB1, DDR1, DDR2, DNMT3A, DOT1L, EGFR, EPHA3, ERBB2, ERBB3, ERBB4, ERCC2, ERG, ERRFI1, ESR1, ETV1, ETV4, ETV5, ETV6, EWSR1, EZH2, FBXW7, FGFR1, FGFR2, FGFR3, FGFR4, FLCN, FLT1, FLT3, FLT4, FOXL2, GNA11, GNAQ, GNAS, HDAC9, HGF, HRAS, IDH1, IDH2, IGF1R, IGF2, JAK1, JAK2, JAK3, KDR, KIT, KMT2A, KRAS, MAP2K1, MAP2K2, MAP2K4, MAP3K1, MAP3K4, MAPK1, MAPK3, MAPK8, MCL1, MDM2, MDM4, MED12, MEN1, MET, MITF, MLH1, MPL, MSH2, MSH6, MTOR, MYC, MYCN, MYD88, NF1, NF2, NFKBIA, NKX2-1, NOTCH1, NOTCH2, NOTCH3, NOTCH4, NPM1, NRAS, NTRK1, NTRK2, NTRK3, NUTM1, PDGFB, PDGFRA, PDGFRB, PIK3CA, PIK3CB, PIK3CD, PIK3R1, PIK3R2, POLE, PPARG, PTCH1, PTEN, RAB35, RAD50, RAF1, RARA, RB1, RET, RHEB, RICTOR, RNF43, ROS1, RSPO1, RSPO2, RUNX1, SMAD2, SMAD4, SMARCA4, SMARCB1, SMO, SRC, STK11, SYK, TET2, TMPRSS2, TOP2A, TP53, TSC1, TSC2, VHL, WT1, XPO1, ZNRF3) to identify genetic mutations in 94 samples from HER2-positive luminal B breast cancer patients. DNA samples were quantified by Qubit 2.0 using dsDNA HS Assay Kit (Life Technologies, Grand Island, NY). Sequencing libraries were prepared with SureSelectXT Library Prep kit (Agilent Technologies, Inc., Santa Clara, California, USA). In brief, 200 ng of genomic DNA from the FFPE samples was fragmented by the Covaris E220 instrument (Covaris, Woburn, MA), and subsequently subjected to end repair, A-tailing and adapter ligation. Unligated adaptors were removed by Agencourt AMPure XP beads (Beckman Coulter, Beverly, MA). The resulting libraries were PCR-amplified and purified with Agencourt AMPure XP beads. Captured libraries were PCR-amplified using Illumina p5 and p7 primers and purified with Agencourt AMPure XP beads. The library was quantified using KAPA Library Quantification kit (KAPA Biosystems), and its fragment size was analyzed by the Bioanalyzer 2100 (Agilent Technologies, Cedar Creek, TX). Once ready, libraries were sequenced on the Illumina HiSeq2500 platforms (Illumina, San Diego, CA)

**Identification of somatic mutations**

FastQC v0.11.5 (http://www.bioinformatics.babraham.ac.uk/projects/fastqc/) software was used for FASTQ file quality control. The adapter sequences were removed by cutadapt v1.9.1.^1^ Sequencing reads were mapped to Human Genome version 19 (hg19) using the Burrows-Wheeler Aligner.^2^ Poorly mapped reads that have a mapping quality (MAPQ) below 20 were removed using Samtools v.1.3.1.^3^ Local realignment around indels and base quality score recalibration were applied with the Genome Analysis Toolkit (GATK 3.4.0).^4^ Duplicated reads were discarded using Picard MarkDuplicates v2.2.4 (https://broadinstitute.github.io/picard/). Somatic mutations including single nucleotide variants (SNVs), small insertions and deletions (INDELs) were identified using MuTect2 algorithm.^5^ False positive variant calls originating from oxoG artifacts were excluded. All of the variants were annotated using SnpEff & SnpSift v4.3,^6^ VEP^7^ and oncotator.^8^ To identify a high-confidence list of putative somatic mutations, the following filtering steps were applied: (1) total allele count >=50 and variant allele frequency >=5%; (2) minor allele frequency (MAF) <1% in gnomAD^9^ All and east Asian (EAS), and 1000 genome project;^10^ (3) nonsynonymous SNVs or indels in coding regions; (4) exclude variants with ‘benign’ or ‘likely-benign’ of ClinVar clinical significance value. Copy number variations (CNVs) were detected using MuTect2 with default parameters. Fifty non-cancer samples (in-house data) were used as panel of normal.

**Reference**

1. Martin M. Cutadapt removes adapter sequences from high-throughput sequencing reads. EMBnet j. 2011 May 2;17(1):10.

2. Li H, Durbin R. Fast and accurate short read alignment with Burrows-Wheeler transform. Bioinformatics. 2009 Jul 15;25(14):1754–1760.

3. Li H, Handsaker B, Wysoker A, Fennell T, Ruan J, Homer N, et al. The Sequence Alignment/Map format and SAMtools. Bioinformatics. 2009 Aug 15;25(16):2078–2079.

4. Van der Auwera GA, Carneiro MO, Hartl C, Poplin R, Del Angel G, Levy-Moonshine A, et al. From FastQ data to high confidence variant calls: the Genome Analysis Toolkit best practices pipeline. Curr Protoc Bioinformatics. 2013 Oct 15;11(1110):11.10.1–11.10.33.

5. Cibulskis K, Lawrence MS, Carter SL, Sivachenko A, Jaffe D, Sougnez C, et al. Sensitive detection of somatic point mutations in impure and heterogeneous cancer samples. Nat Biotechnol. 2013 Mar;31(3):213–219.

6. Cingolani P, Platts A, Wang LL, Coon M, Nguyen T, Wang L, et al. A program for annotating and predicting the effects of single nucleotide polymorphisms, SnpEff: SNPs in the genome of *Drosophila melanogaster* strain w1118; iso-2; iso-3. Fly (Austin). 2012 Jun;6(2):80–92.

7. McLaren W, Gil L, Hunt SE, Riat HS, Ritchie GRS, Thormann A, et al. The ensembl variant effect predictor. Genome Biol. 2016 Jun 6;17(1):122.

8. Ramos AH, Lichtenstein L, Gupta M, Lawrence MS, Pugh TJ, Saksena G, et al. Oncotator: cancer variant annotation tool. Hum Mutat. 2015 Apr;36(4):E2423–9.

9. Lek M, Karczewski KJ, Minikel EV, Samocha KE, Banks E, Fennell T, et al. Analysis of protein-coding genetic variation in 60,706 humans. Nature. 2016 Aug 18;536(7616):285–291.

10. 1000 Genomes Project Consortium, Auton A, Brooks LD, Durbin RM, Garrison EP, Kang HM, et al. A global reference for human genetic variation. Nature. 2015 Oct 1;526(7571):68–74.
