# Supplementary material for: High polymerase ε expression associated with increased CD8+T cells improves survival in patients with non-small cell lung cancer
Source: PLoS One. 2020 May 20;15(5):e0233066. doi: 10.1371/journal.pone.0233066 (PMC7239475; doi:10.1371/journal.pone.0233066)
Supplement: S1 Table — (DOCX) [file pone.0233066.s003.docx]

**S1 Table** Gene mutation profiles in non-small cell lung cancer in the KUMC cohort

| Gene | Wild | Mutant | Percentage |
| --- | --- | --- | --- |
| POLE | 71 | 97 | 57.74% |
| EGFR | 93 | 75 | 44.64% |
| TP53 | 96 | 72 | 42.86% |
| MED12 | 99 | 69 | 41.07% |
| PIK3CA | 145 | 23 | 13.69% |
| MSH6 | 145 | 23 | 13.69% |
| KRAS_1 | 147 | 21 | 12.50% |
| MLH1 | 152 | 16 | 9.52% |
| ATR | 152 | 16 | 9.52% |
| APC | 154 | 14 | 8.33% |
| JAK2 | 155 | 13 | 7.74% |
| ATM | 155 | 13 | 7.74% |
| RAD50 | 155 | 13 | 7.74% |
| BRCA2 | 156 | 12 | 7.14% |
| MET | 157 | 11 | 6.55% |
| BRCA1 | 157 | 11 | 6.55% |
| CDH1 | 158 | 10 | 5.95% |
| TSC1 | 159 | 9 | 5.36% |
| ALK | 160 | 8 | 4.76% |
| PTCH1 | 160 | 8 | 4.76% |
| PTEN | 160 | 8 | 4.76% |
| CTNNB1 | 160 | 8 | 4.76% |
| RET | 160 | 8 | 4.76% |
| CDKN2A | 161 | 7 | 4.17% |
| BRAF | 161 | 7 | 4.17% |
| CHEK2 | 161 | 7 | 4.17% |
| NF1 | 161 | 7 | 4.17% |
| RB1 | 163 | 5 | 2.98% |
| JAK3 | 163 | 5 | 2.98% |
| ERBB2 | 164 | 4 | 2.38% |
| TSC2 | 164 | 4 | 2.38% |
| PDGFR | 164 | 4 | 2.38% |
| MSH2 | 165 | 3 | 1.79% |
| NOTCH1 | 165 | 3 | 1.79% |
| KIT | 165 | 3 | 1.79% |
| SMARCA4 | 165 | 3 | 1.79% |
| CDK4 | 166 | 2 | 1.19% |
| VHL | 166 | 2 | 1.19% |
| RUNX1 | 166 | 2 | 1.19% |
| ERBB | 166 | 2 | 1.19% |
| DDR2 | 166 | 2 | 1.19% |
| DNMT3A | 166 | 2 | 1.19% |
| FGFR2 | 166 | 2 | 1.19% |
| FLCN | 166 | 2 | 1.19% |
| RAF1 | 166 | 2 | 1.19% |
| MAP2K1 | 166 | 2 | 1.19% |
| ASXL1 | 166 | 2 | 1.19% |
| PIK3R1 | 167 | 1 | 0.60% |
| PIK3R2 | 167 | 1 | 0.60% |
| MSH1 | 167 | 1 | 0.60% |
| WT1 | 167 | 1 | 0.60% |
| MPL | 167 | 1 | 0.60% |
| FBXW7 | 167 | 1 | 0.60% |
| ERCC2 | 167 | 1 | 0.60% |
| NOTCH3 | 167 | 1 | 0.60% |
| MYC | 167 | 1 | 0.60% |
| STK11 | 167 | 1 | 0.60% |
| ERBB3 | 167 | 1 | 0.60% |
| AKT1 | 167 | 1 | 0.60% |
| JAK | 167 | 1 | 0.60% |
| CTTNB1 | 167 | 1 | 0.60% |
| NOTCH4 | 167 | 1 | 0.60% |
| XPO1 | 167 | 1 | 0.60% |
| STK1 | 167 | 1 | 0.60% |
| FGFR1 | 167 | 1 | 0.60% |
| FGFR3 | 167 | 1 | 0.60% |
| SMAD4 | 167 | 1 | 0.60% |
| KDR | 167 | 1 | 0.60% |
| IDH2 | 167 | 1 | 0.60% |
| ETV6 | 167 | 1 | 0.60% |
| PDGFRA | 167 | 1 | 0.60% |
| PDGFRB | 167 | 1 | 0.60% |
| CREBBP | 167 | 1 | 0.60% |
| MAP2K2 | 167 | 1 | 0.60% |
| GNAS | 167 | 1 | 0.60% |
