# Supplementary material for: High polymerase ε expression associated with increased CD8+T cells improves survival in patients with non-small cell lung cancer
Source: PLoS One. 2020 May 20;15(5):e0233066. doi: 10.1371/journal.pone.0233066 (PMC7239475; doi:10.1371/journal.pone.0233066)
Supplement: S2 Table — (DOCX) [file pone.0233066.s004.docx]

**S2 Table** Protein sequence changes in the POLE mutants in the KUMC cohort

| Protein changes | Frequency | Percent |
| --- | --- | --- |
| wild type | 71 | 42.26% |
| frame_shift_p.V1446fs | 89 | 52.98% |
| frame_shift_p.V1446fs , p.E719K_missense variant | 1 | 0.60% |
| frame_shift_p.V1446fs , p.R2259Q_missense variant | 1 | 0.60% |
| frame_shift_p.V1446fs, p.R1273L_missense variant | 1 | 0.60% |
| frame_shift_p.V1446fs, p.R1320Q_missense variant | 1 | 0.60% |
| frame_shift_p.V1446fs, p.R1634H_missense variant | 1 | 0.60% |
| frame_shift_p.V1446fs, p.R52W_missense variant | 1 | 0.60% |
| p.A1866T_missense variant | 1 | 0.60% |
| p.R1634H_missense variant, p.R1382H_missense variant | 1 | 0.60% |
