# Supplementary material for: High polymerase ε expression associated with increased CD8+T cells improves survival in patients with non-small cell lung cancer
Source: PLoS One. 2020 May 20;15(5):e0233066. doi: 10.1371/journal.pone.0233066 (PMC7239475; doi:10.1371/journal.pone.0233066)
Supplement: S3 Table — (DOCX) [file pone.0233066.s005.docx]

**S3 Table** Eleven KEGG gene sets associated low POLE expression levels (TCGA data)

| Name | Size | ES | NES | NOM  (p-value) | FDR  (q-value) | FWER  (p-value) |
| --- | --- | --- | --- | --- | --- | --- |
| KEGG_CELL_CYCLE | 118 | 0.673 | 2.278 | <0.001 | 0.002 | 0.001 |
| KEGG_SPLICEOSOME | 114 | 0.675 | 2.192 | <0.001 | 0.003 | 0.004 |
| KEGG_HOMOLOGOUS_RECOMBINATION | 26 | 0.786 | 2.090 | <0.001 | 0.005 | 0.016 |
| KEGG_DNA_REPLICATION | 35 | 0.793 | 1.963 | 0.002 | 0.021 | 0.072 |
| KEGG_MISMATCH_REPAIR | 23 | 0.715 | 1.911 | 0.006 | 0.032 | 0.13 |
| KEGG_PROGESTERONE_MEDIATED_OOCYTE_MATURATION | 85 | 0.471 | 1.895 | 0.002 | 0.032 | 0.157 |
| KEGG_BASE_EXCISION_REPAIR | 32 | 0.617 | 1.868 | 0.008 | 0.036 | 0.192 |
| KEGG_LYSINE_DEGRADATION | 44 | 0.505 | 1.799 | 0.010 | 0.058 | 0.304 |
| KEGG_OOCYTE_MEIOSIS | 112 | 0.436 | 1.790 | 0.002 | 0.056 | 0.319 |
| KEGG_BASAL_TRANSCRIPTION_FACTORS | 35 | 0.512 | 1.658 | 0.032 | 0.132 | 0.602 |
| KEGG_RNA_DEGRADATION | 57 | 0.482 | 1.633 | 0.047 | 0.142 | 0.662 |

ES, enrichment score; NES, normalized enrichment score; FDR, false discovery rate; NOM, nominal p-value; FDR, false discovery rate; FWER, family-wise error rate
