# Supplementary material for: High polymerase ε expression associated with increased CD8+T cells improves survival in patients with non-small cell lung cancer
Source: PLoS One. 2020 May 20;15(5):e0233066. doi: 10.1371/journal.pone.0233066 (PMC7239475; doi:10.1371/journal.pone.0233066)
Supplement: S4 Table — (DOCX) [file pone.0233066.s006.docx]

**S4 Table** Immune gene sets associated with high POLE expression levels (TCGA data)

| Name | Size | ES | NES | NOM  (p-value) | FDR  (q-value) | FWER  (p-value) |
| --- | --- | --- | --- | --- | --- | --- |
| REACTOME_ANTIGEN_PRESENTATION_FOLDING_ASSEMBLY_AND_PEPTIDE_LOADING_OF_CLASS_I_MHC | 15 | 0.651 | 1.594 | 0.044 | 0.183 | 0.116 |
| GO_ANTIGEN_PROCESSING_AND_PRESENTATION_OF_PEPTIDE_ANTIGEN_VIA_MHC_CLASS_I | 84 | 0.327 | 1.130 | 0.330 | 0.794 | 0.547 |
| KEGG_ANTIGEN_PROCESSING_AND_PRESENTATION | 63 | 0.266 | 0.871 | 0.594 | 1.000 | 0.802 |
| GO_ANTIGEN_RECEPTOR_MEDIATED_SIGNALING_PATHWAY | 161 | 0.200 | 0.770 | 0.717 | 1.000 | 0.862 |
| GO_INFLAMMATORY_RESPONSE_TO_ANTIGENIC_STIMULUS | 23 | 0.223 | 0.727 | 0.849 | 0.930 | 0.887 |

ES, enrichment score; NES, normalized enrichment score; FDR, false discovery rate; NOM, nominal p-value; FDR, false discovery rate; FWER, family-wise error rate
