# Supplementary material for: High polymerase ε expression associated with increased CD8+T cells improves survival in patients with non-small cell lung cancer
Source: PLoS One. 2020 May 20;15(5):e0233066. doi: 10.1371/journal.pone.0233066 (PMC7239475; doi:10.1371/journal.pone.0233066)
Supplement: S5 Table — (DOCX) [file pone.0233066.s007.docx]

**S5 Table** Pearson's correlation between high POLE expression levels and endogenous retroviral elements

| Endogenous retrovirus | Pearson's correlation |
| --- | --- |
| ERV9-1 | 0.327 |
| ERVW-1 | 0.296 |
| ERVV-1 | 0.282 |
| ERVV-2 | 0.267 |
| ERVK-22 | 0.250 |
| ERVK-21 | 0.245 |
| ERVK-6 | 0.244 |
| ERVK-1 | 0.227 |
| ERVW-4 | 0.218 |
| ERVH-1 | 0.215 |
| ERVFRD-2 | 0.214 |
| ERVH-3 | 0.214 |
| ERVS71-2 | 0.198 |
| ERVMER61-1 | 0.195 |
| ERVH-2 | 0.194 |
| ERVH-7 | 0.188 |
| ERVH-4 | 0.188 |
| ERVK3-4 | 0.177 |
| ERVH-5 | 0.176 |
| ERVH-6 | 0.174 |
| ERVFH21-1 | 0.171 |
| ERVW-5 | 0.152 |
| ERVFC1-1 | 0.152 |
| ERVW-3 | 0.149 |
| ERVK3-5 | 0.126 |
| ERVK-7 | 0.120 |
| ERVK3-7 | 0.100 |
| ERVH48-1 | 0.096 |
| ERVK-15 | 0.095 |
| ERVE-4 | 0.094 |
| ERVK3-2 | 0.093 |
| ERVK3-8 | 0.085 |
| ERVS71-1 | 0.085 |
| ERVK-23 | 0.084 |
| ERVK-16 | 0.080 |
| ERV3-2 | 0.077 |
